# “Helping someone with a skill sharpens it in your own mind”: a mixed method study exploring health professions students experiences of Peer Assisted Learning (PAL)

**DOI:** 10.1186/s12909-016-0566-8

**Published:** 2016-02-04

**Authors:** Sandra E. Carr, Gabrielle Brand, Li Wei, Helen Wright, Pam Nicol, Helene Metcalfe, Julie Saunders, John Payne, Liza Seubert, Laurie Foley

**Affiliations:** Faculty of Medicine, Dentistry and Health Sciences, The University of Western Australia, MB515, 35 Stirling Hwy, Crawley, 6009 WA Australia

**Keywords:** *Peer assisted learning*, *Peer feedback*, *Collaborative learning*, *Peer teaching*, *Health professions education*

## Abstract

**Background:**

Peer assisted learning (PAL) has been described as “the development of knowledge and skill through active help and support among status equals or matched companions”. To enhance the learning experience of health professions students and improve collaborative and collegial learning, six pilot Peer Assisted Learning (PAL) projects were conducted across a health science faculty.

**Methods:**

A responsive mixed method evaluation design was applied to explore the adequacy of the preparation for PAL, the impact PAL had on student attainment of examination, consultation, communication and feedback skills and to explore students’ learning experiences through PAL.

**Results:**

The 149 participants agreed the training programme was well organised, offered a safe learning environment and prepared the participant for the PAL activity. The impact of PAL included improvements in students’ confidence and ability to give feedback and developed students’ teaching, clinical and communication skills. Qualitative analysis revealed participants experienced deeper learning through teaching and learning from their peers, became more open to giving and receiving feedback and valued the comfortable/safe learning environment offered through PAL.

**Conclusion:**

Providing appropriate training in peer teaching and feedback and the schools engagement and openness to peer learning in the classroom and clinical setting enhances students’ peer assisted learning experience.

## Background

Peer assisted learning (PAL) has been described as “the development of knowledge and skill through active help and support among status equals or matched companions” [1]. The benefits of PAL have been reported to positively correlate with examination performance, to lower student distress and enhance course satisfaction through the establishment of a reciprocal social support system [2, 3]. PAL has been utilised increasingly in higher education of health professionals to support increased early clinical contact and as part of formal education programmes acting as a vehicle to enhance students’ learning experience and skills [4–6]. Many of these projects have attempted to enhance students’ clinical skills, systematic examination skills, mentoring or teaching skills, cognitive ability, psychomotor development, and social integration [6–8]. The rise in global interest in using PAL within higher degree education is largely due to the increasing expectations for health professions graduates to achieve competency and experience in teaching and feedback [9]. All of which are identified in key statements by both the United Kingdom’s General Medical Council (GMC) [10] and the Australian Medical Council (AMC) [11]. However, more mixed methods and particularly qualitative research [12, 13] has been called for to explore the process of PAL in more depth and describe the intangible factors that influence health profession’s students PAL learning experiences in different settings.

In a commitment to enhance the learning experience of health professions students, and promote strategic pedagogical change that enhances collaborative and collegial learning, the University of Western Australia’s (UWA) Faculty of Medicine, Dentistry and Health Science (FMDHS) conducted six pilot PAL projects across the Faculty in 2013. Prior to this, PAL activities across the faculty were informal, external to curricula and ad hoc in approach. One of the main aims of the project was to formally implement and integrate PAL activities into the health professional courses in a systematic and sustainable manner, across both the classroom and clinical settings. The PAL project outcomes were to:

- Develop a training programme for students on peer teaching and feedback.
- Develop teaching resources to assist the implementation of peer assisted learning.
- Develop tools to evaluate PAL
- Describe evaluation findings for the six pilot projects

### PAL project outline

In semester 1, 2013, six main areas for learning development across the faculty that could be enhanced through PAL were identified. These included PAL in communication, consultation and clinical skills development in the clinical setting and referencing, critical writing and case presentation skills in the classroom setting. Following UWA Human Research Ethics Committee approval (RA/4/1/6127), the PAL planning framework for the pilot PAL activities across six health disciplines including medicine, nursing, podiatric medicine, pharmacy, dentistry and health science was finalised (see Table 1).

**Table 1:** Summary of Pilot PAL projects at UWA

Course (no. in cohort)	Programme	Student level involved	Setting	Number of students (*N* = 149)
Medicine-Paediatric	Peer tutoring on paediatric clinical examination skills	Year 5 as “peer learners”	clinical	Year 5 cohort (*n* = 41)
Y5 (120)		Year 6 as “peer tutors”		Year 6 cohort (*n* = 12)
Y6 (180)				
Nursing	Peer tutoring on clinical communication skills	Year 1 as “peer learners”	clinical	Year 1 cohort (*n* = 4)
Y1 (35)		Year 2 as “peer tutors”		Year 2 cohort (*n* = 5)
Y2 (10)				
Podiatric Medicine	Peer tutoring ON clinical consultation skills	Year 3 as “peer learners”	clinical	Year 3 cohort (*n* = 3)
Y3 (40)		Year 4 as “peer tutors”		Year 4 cohort (*n* = 3)
Y4 (30)				
Pharmacy (*N* = 30)	Peer verbal feedback on referencing and critical writing skills	Year 2 as “peer assessors”	class room	Year 2 cohort (*n* = 28)
Health sciences (*N* =40)	Peer written feedback on developing data collection tool	Year 3 as “peer assessors”	class room	Year 3 cohort (*n* = 22)
Dentistry (*N* = 45)	Peer feedback on case presentation	Year 4 as “peer assessors”	class room	Year 4 cohort (*n* = 31)

At the beginning of Semester 2, 2013 a training programme for the peer learners/tutors was developed. This targeted the specific skills required to facilitate an effective outcome for each PAL activity. In the clinical setting, the peer tutors were recruited following an expression of interest in becoming a PAL tutor [14] and were identified as suitable by the unit coordinator. Of the twenty one students who volunteered for PAL, twenty completed the PAL training, with one podiatric medicine student withdrawing due to other study commitments. Within the classroom setting, the PAL activities were embedded as a whole-class approach within the health professions unit’s content and delivered in timetabled face-to-face tutorial time.

Each training session covered the potential benefits of PAL, the specifics of the skill being taught and/or learned during PAL activity (clinical examination, consultation, communication, critical writing, presentation) and an opportunity to interact, develop and practise giving constructive feedback to each other (written and/or verbal). An open discussion about issues and topics which may emerge through PAL experiences, including expectations (roles and behaviour) was also encouraged during the training sessions [15]. In collaboration with each health professions unit coordinators, PAL training was conducted in face to face settings for between 1 to 4 h and was delivered by the 1st (SC), 4th (HW) and 5th (PN) authors of the PAL project team.

This article presents the evaluation findings of the pilot PAL projects and includes a discussion of the methodological issues and practical implications that arose.

## Methods

The PAL activities were evaluated using a responsive mixed method evaluation design to answer the following questions:

1. Was the training programme relevant and adequate to prepare students for the PAL activities?
2. What impact did the PAL have on student attainment of examination, consultation, communication and feedback skills?
3. What were the students’ learning experiences through PAL?

Due to the multiple PAL project aims, a mixed method of outcome evaluation was used [16, 17]. The PAL evaluation tools were designed to constructively align [20] with the PAL project aims and the PAL activity’s specific learning objectives.

Both quantitative and qualitative data was collected over a period of 14 weeks to coincide with the timing of the academic semester. Data collection consisted of six sources:

1. Pre PAL survey of each of the six pilot projects.
2. Survey to evaluate the training programmes.
3. Direct observation of PAL activity in action for each of six pilot projects.
4. Post PAL survey.
5. Post PAL focus group discussion/interview with students.
6. Post PAL focus group discussion with staff (November 12th).

The participants for this study were a purposeful sample drawn from 149 health professional students across six courses in the Faculty of Medicine, Dentistry and Health Sciences at The University of Western Australia. Written consent was obtained from each participant in face to face settings prior to the project commencing. Participation was voluntary and all survey instruments were coded to de-identify responses.

The quantitative data collection survey tool focused on gathering participants’ attitudes of PAL, before and after engaging in the PAL training programme and PAL activity, and the influence of PAL on skill attainment and to gather suggestions for improvement to PAL process. The survey instruments were developed for each pilot project area by the project team and used 4 point Likert Scale type items (1 = Strongly disagree, 2 = disagree, 3 = agree, 4 = strongly agree) and included questions such as “*My peer instructor gave me effective verbal feedback on my clinical skills”* and *The PAL teaching was a safe learning environment*.”

The qualitative data collection was grounded in phenomenology as the focus was on capturing the health profession’s student’s experiences of PAL within the clinical and classroom settings. This was achieved by firstly, the 2nd author (GB) capturing 10 periods (*n* = 16 h) of observational data through field notes in both the training and in classroom and clinical setting during the PAL activities. The bank of observational data collected during the PAL training and observation of PAL activities was used to inform selection of the reflective questioning prompts used during the focus groups and interview process. Students and staff were then invited to participate in an audio-recorded focus groups (*n* = 7) and/or interviews (n-9) conducted by the first two authors (SC; GB) to explore their experiences of PAL in more depth. Credibility of the data was determined by the researchers summarising and debriefing directly after focus/groups and/or interviews to ensure accuracy of the researcher’s interpretation of what the participants were saying. The female authors who collected the data both have a PhD and experience in qualitative research and are not involved in the teaching or coordination of any units from the research sample. Thus, there was no potential dependent relationship identified between interviewer and participants.

### Data analysis

A range of approaches were used to analyse the data collected. For quantitative data, descriptive statistics were calculated to report the response rates for each of the data collection points and to summarise the survey data. To enable a comparison of survey data for Pre and Post PAL a Wilcoxon signed ranks test was used to compare paediatric students’ confidence in clinical examination skills and interaction with children prior to and after peer tutoring. A Mann Whitney *U* test or a Kruskal Wallis test was undertaken to compare survey responses from both the clinical and classroom settings about the training, experience of PAL and impact on learning.

Preliminary qualitative analysis by the first two authors (SC; GB) commenced early during the training and observational periods, guiding the interpretative lens and informing later analysis. Emergent thematic analysis [18] was used by the first three authors (SC; GB; LW) to search for larger patterns, congruency, meanings and points of thematic convergence that cut across the survey, observational field notes, focus group and interview data. This ensured triangulation, as emerging themes were derived from the surveys, interview and observational data sources.

Ideas and key concepts were identified condensing the data set into units for analysis and a coding tree was used as a method of organizing the large amount of data and was part of the analytical process because giving codes to data and developing concepts, enables a rigorous evaluation of what the data was saying [19]. The back and forth process between data sources confirmed the original concepts, endorsing the initial themes as being representative of the data. This analysis process continued until saturation occurred and the themes were distilled and captured into overarching themes within the different PAL settings.

## Results

### Quantitative findings

Table 1 offers an overview of the PAL pilot research course, programme, student level, learning setting and student numbers. Participant demography included 48 male and 89 female student respondents (missing data 12), aged between 19 to 41 years, with a mean age of 23.3 years.

The researchers engaged in 8 h of observation in each clinical and classroom settings, culminating in 10 periods of observation and collecting 16 h of observational data in total

In the clinical setting, interview and focus group data were collected from 8 peer tutors, and 14 peer learners. In the classroom setting, data were collected from 8 students in 2 focus groups.

### Training programme evaluation

Overall, respondents highly agreed (86 to 100 %) the training programme conducted in the six different pilots was “well organised”, “offered a safe learning environment” and provided sufficient and helpful information in preparing the participant for the PAL activity (Tables 2, 3 and 4).

**Table 2:** Satisfaction with Pre-PAL Training Programme Evaluation in the Classroom setting

	Dentistry (*N* = 31)		Health Sciences (*N* = 22)		Pharmacy (*N* = 28)	
Items	Mean	Agreement (%)	Mean	Agreement (%)	Mean	Agreement (%)
Q3_well_organized	3.14	100	3.38	100	3.23	92.4
Q4_safe_environment*	3.34	100	3.68	100	3.62	100
Q5_sufficient_information*	3.03	86.2	3.45	95.5	3.40	100
Q6_helpful_information**	3.11	96.4	3.68	100	3.58	100
Q7_engagement	3.89	88.9	3.9	90.5	4.00	100

*p < .05

**p < .01

**Table 3:** Satisfaction with Pre-PAL Training Programme in Clinical setting (peer tutors)

Items	Paediatric (*n* = 12)		Nurse & Podiatric (*n* = 8)	
	Mean	Agreement (%)	Mean	Agreement (%)
Q3_info_preparation	3.25	91.6	3.38	100
Q4_well_organization	3.67	100	3.88	100
Q5_safe_environment	3.67	100	3.88	100
Q6_refresh_skills	3.67	100	3.00	100
Q7_info_teaching	3.67	100	3.38	100
Q8_confidence_meds	3.67	100	3.50	100
Q9_confidence_feedback	3.75	100	3.43	100
Q10_useful_feedback	3.25	91.6	3.43	100
Q11_prepare_being_tutor	3.58	100	3.29	100
Q12_engagement	4.00	100	4.00	100

**Table 4:** Confidence in skills Pre-PAL in Clinical setting (peer learners)

Items	Paediatric (*n* = 41)		Nurse & pod (*n* = 7)	
	Mean	Agreement (%)	Mean	Agreement (%)
Q2_confidence_exam_skills	1.85	4.9	3.0	100
Q3_confidence_consultation_skill	1.88	7.3	3.0	100
Q4_confidence_interaction	2.85	78.1	3.0	100

### Influence of PAL on skill attainment

In summary, the quantitative findings relating to the impact of the PAL programmes demonstrated a high proportion of respondents agreeing that PAL had improved their confidence and ability to give feedback (95 to 100 % agreement). All pharmacy and health science students valued the feedback they had received and a high proportion agreed their peers gave verbal and written feedback effectively. Between 85 and 90 % of respondents in the classroom settings, agreed that PAL had met their initial expectations.

Evaluation of the PAL activities by the peer tutors in clinical settings demonstrates close to 100 % agreement across the three courses (medicine, nursing and podiatry) that they provided a safe environment, clear instructions, and that the learner improved as a result of their peer teaching. All agreed that the PAL experience had increased their teaching skills, met their expectations and that they would recommend being a PAL tutor to future students.

A Wilcoxon signed rank test was conducted to compare clinical peer learners’ confidence in examination skills and interaction with children pre and post PAL. Interestingly, in the clinical setting, there was an increase in clinical peer learners’ (from medicine) confidence in paediatric examination and consultation skills with children pre and post PAL activity. The results indicated that PAL had significantly greater relative influence on clinical examination skills amongst paediatric (medicine) students after peer tutoring, *T* = 131.00, *z* = -3.345 (corrected for ties), *N* – Ties = 16, *p* = 0.001 (two-tailed), and this effect can be considered “large”, *r* = 0.84.

### Qualitative findings

**Fig. 1:**
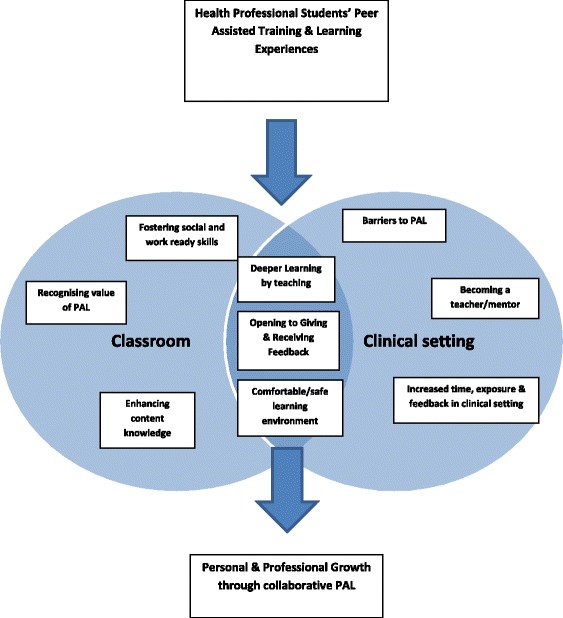
Health professional students’ experiences of PAL

The themes that captured the health professional students’ PAL experiences are presented in a Venn diagram (see Fig. 1) with two overlapping circles to signpost the students’ experiences of PAL in the classroom and the clinical setting. The intersection of the two circles represents the three shared PAL themes experienced in both the classroom and the clinical setting. The nine major themes are briefly described with supportive quotations from qualitative survey responses, interview and focus group transcripts.

### Shared PAL Qualitative Themes from the classroom and clinical setting

The three major themes that emerged from the PAL qualitative data across the classroom and clinical setting were explored:

- Deeper learning by teaching.
- Opening to giving and receiving feedback.
- Comfortable/safe learning environment.

- Deeper learning by teaching

A shared theme across all of the data was that PAL presented an opportunity to widen and deepen the student’s learning experience by teaching and giving feedback. As one nursing student articulated helping someone with a skill “*sharpens it in your own mind”.* The majority of students expressed how teaching and giving feedback to their peers helped conceptualize their understanding by reflecting on their own learning needs and bridging the gap between theory and practice:

*It made you critically review your own work like when you were giving feedback to other people it would make you critically like review your own work as well which was kind of good….(health science student).*

*It gave me the opportunity to do sort of reflection, self-reflection a lot. I was looking at you know myself, stuff that I do through another eye… (nursing student tutor).*

b) Opening to giving and receiving feedback

All of the students described how the PAL activities assisted them in learning how to give and receive peer feedback. Following the PAL activity, many of the students began to explore their expectations of PAL and how this would impact on their learning:

*being an active listener during feedback and if asking for feedback, to be specific, accept it and not be defensive (pharmacy student).*

Many of the students described how peer feedback is more honest, constructive and ‘*a bit more nitty gritty’* and in many cases easier to receive because ‘*it is a less stressful feeling receiving feedback from peers, helps because more common ground is shared’.*

The PAL research highlighted how giving and receiving feedback from the same or near peer level is based on relatedness and a shared understanding of each other’s learning needs. This was highlighted in the following student quote:

*it provided a fresh pair of eyes and constructive feedback on the assignment from peers that had the same task and a similar background and expectations. This made it more relaxed and could easily understand each other. Maybe understood more clearly and specifically than feedback from lecturers. Definitely, ways of improvement were more abundant from the peers than from lecturers (health science student).*

A sense of responsibility to each other and the importance of giving constructive and helpful feedback in a sensitive way was expressed by many of the students as they expressed ‘*fear of giving badly structured feedback’* and not ‘*wanting to sound negative*’. This was typified in a student’s quote when asked about some of the challenges of PAL in the classroom:

*Not to hurt their feelings and when you are giving feedback that’s why you go through the things that they did well first. This is a way you could have improved so that you kind of create a safe environment. Like, you weren’t cruel…(health science student).*

c) Comfortable/safe learning environment

One of the most noticeable shared themes to emerge from the data was that PAL provided a safe comfortable learning environment where the students felt they could ask questions and ‘*felt safe to make mistakes’*. One student expressed how learning from a peer was ‘*less intimidating for students…more comfortable to voice opinions and ask questions’,* PAL offered an environment whereby the student *‘can express all their fears or their awkwardness and things like that…’ (nursing student tutor).*

Many of the students talked about how the formative nature of the PAL activity was less stressful and took the pressure off their perceived need to perform:

*it will be more related and without fear of being assessed (podiatry student tutor).*

*I felt I was able to concentrate more on getting things out of the session rather than being stressed about not performing well (medical student learner)*

A preponderance of the students identified that PAL enhanced their learning because they felt safe, understood and comfortable to explore and reflect on their own learning needs:

*when there is more pressure I tend to just like get more nervous and make more mistakes, whereas if it’s just student it just makes me less anxious and nervous and then I can just ask questions, and its more approachable and, I don’t know, easy environment to actually do something in..(podiatry student leaner).*

### PAL qualitative themes from the classroom

The PAL qualitative data from the classroom was drawn from 90 students across three courses in the UWA FMDHS. The major PAL themes identified in the same-ability classroom setting where students experienced being a peer tutor and learner were:

- Recognising the value of PAL
- Fostering social and work ready skills
- Enhancing content knowledge

Most student comments were based around opening to a new way of learning, recognizing that their peers could provide valuable and constructive feedback which enhanced both their theory based unit knowledge and social skills to prepare them for their future health professional roles. This was demonstrated in a student’s comment when asked about the benefits of PAL:

*the ability to critically assess my peers and also receive feedback from my peers to better my education (dentistry student)*

- Recognising the value of PAL

Many student comments included recognizing PAL as a new and different way of learning in the classroom. The peer assisted learning experience offered valuable feedback from a different perspective, an experience they described in their expectations of PAL:

*I hope to develop more skills and find out what attributes are lacking in me. I hope to also see how different people see me (dentistry student)*

*It will give another perspective to an assignment and probably a better interpersonal relationship so feedback is more forthright (health science student).*

b) Fostering social and work ready skills

A major theme from the students’ experiences of PAL included how PAL assisted with developing communication skills required for future professional practice. Student comments included:

*it allowed for more open communication. There was a lot more ease of accepting and giving criticism*

*better ways of providing for future job experience (health science student)*

The opportunity to work in teams and learn from and with each other was another common theme identified by the students:

*Gain good learning opportunities that are valuable as my peers and I am able to figure out problem together (dentistry student).*

The majority of students also described how they appreciated the social interaction PAL offered by presenting an opportunity to ‘*meet other students and network’* and as one student stated: *‘it gave me a chance to talk to my peers and support them’*.

c) Enhancing content knowledge

By engaging in PAL, students reported an improved knowledge of the unit’s content due to increased quantity and quality of peer feedback:

*The feedback was very honest and less condescending. The suggestions are more practical and can be implemented (pharmacy student).*

For some of students, the feedback was more relevant and responsive to their learning level and needs:

*You seem to get another viewpoint from a student perspective and not from a professional viewpoint. It made it seemingly more relevant and more true to life. (pharmacy student)*

However, a few students indicated their caution in receiving feedback from a same level peer, actively questioning the credibility of the feedback they received:

*They were at the same level, I was not confident that their feedback was accurate – felt the need to clarify this with teacher (health science student).*

### PAL Qualitative Themes from the clinical setting

The PAL qualitative data from the clinical setting was drawn from 65 students across three courses in the UWA FMDHS. These included medicine, podiatry and nursing. The major PAL themes identified from the cross-ability clinical setting data collected from peer tutors and peer learners included:

- Increased time, exposure and feedback in the clinical setting
- Becoming a teacher/mentor
- Barriers to PAL

Most of the students’ comments were based around reinforcing and enhancing clinical skills due to increased exposure and time in the clinical setting. The student’s role as peer teacher or peer learner enhanced their interpersonal and communication skills through a learning exchange that encouraged interaction and inquiry. This was demonstrated in a student’s comment when asked about the benefits of PAL in the clinical setting:

*greater confidence in my clinical skills with children and other patients (medical student tutor).*

*we could get on with it without any pressure and then receive feedback for that was constructive but formative..(medical student learner)*

- Increased time, exposure and feedback in the clinical setting

The increased time, exposure and feedback on specific clinical skills increased both peer tutor and learner’s knowledge and clinical skills in a safe learning environment:

*when you are teaching you also pick up points on the examination and it forces you to analyse like why do I look for those particular signs or how much value do I get from eliciting those signs or you know. You know I think you do refine your own clinical skills, and your own knowledge as well and you go and do a bit more reading up on certain things that you might not be sure of, or questions that students have asked (medical student tutor).*

The feedback received from the peer tutors was both relevant and meaningful because the peer tutors had recently gone through the same experience:

*Feedback was very level appropriate. Since the students had only done their paediatric term the previous year they knew what we had to learn during our time here (medical student learner)*

*gone through it more recently, a bit more understanding of how we feel being the students going through the learning (medical student learner).*

From the peer tutor perspective, an advantage of PAL included the opportunity to reflect on their own learning and practice as indicated in this comment by a podiatry peer tutor:

*watching the other students interact with the patients because if we are with other students in the cubicle we will tag team we both sort of consult the patient, you know. In this session we could sit back and watch a bit more and I think watching them interact with the patient sort of gave me some not feedback but gave me some things to think about when I see patients (podiatry student tutor).*

b) Becoming a teachers/mentor

The health professional students volunteered to be PAL tutors in the clinical setting, had already indicated a previous positive experience of PAL or a strong interest in teaching which was consequently enhanced following the PAL activity:

*That I really enjoy teaching and that it’s something that I want to keep doing (6th year medical student)*

For some peer tutors, the PAL experience gave them an opportunity to engage with peer learners in an informal social setting, whereby the learning was reciprocal and more organic:

*Informal relationship being a friend or mentor…It’s more comfortable and induces a more conducive learning environment as opposed to a more formal one (podiatry student tutor)*

Many of the peer tutors enjoyed the opportunity to discuss their previous course and clinical experiences, becoming a valued mentor:

*I enjoyed being able to interact with students from the year below and discuss issues not just pertinent to the PAL programme, but to medicine in general. Have been mentoring one student as a result since my involvement in the programme (medical student tutor).*

c) Barriers to PAL

Although the majority of students had a positive experience of PAL in the clinical setting, some of the students expressed the importance of organisational structures to ensure the PAL activities ran smoothly:

*I thought the hardest thing about the whole thing was just organising the session at a mutual time and date (medical student tutor).*

Some peer tutors also commented on the importance of clarifying the peer learner’s expectations prior to the PAL exchange:

*It was sometimes difficult as the learners had unrealistic expectations (nursing student tutor)*

## Discussion

The purpose of this research study was to develop, implement and evaluate six pilot projects focused on Peer Assisted Learning (PAL) across a Health Sciences Faculty over a 6 month period. Using a mixed methodology, the study has been able to achieve the main aims by successfully evaluating the effectiveness of PAL training, the influence of PAL on skill attainment and gain a deeper understanding of students’ experiences of PAL in the classroom and clinical setting. The findings from this study present an argument for the implementation of PAL as an effective teaching and learning tool in health professional student training and education.

These findings are consistent with current PAL literature that suggests PAL enhances student learning experience in higher education across the classroom and clinical setting by providing valuable teaching experience and enhancing their ability to give and receive constructive feedback, a key element in instilling a culture of teaching in future health professionals [9]. As described in the findings, the PAL activities enhanced learning by consolidating content and clinical knowledge and skills through participation, cooperative informal learning [7, 20] and provides a reciprocal social support system for both peer tutor and peer learner [21–23].

Many students expressed how teaching and giving feedback to their peers in an informal learning environment lowered their stress level, helped conceptualise their understanding, providing them with an opportunity to comfortably explore and express their ideas, thoughts and questions and reflect on what they have learned. This concept of PAL is based on social learning theory and advocates collaborative and active learning. As Topping stated “PAL can lead to generalization from the specific situated example through which a concept is learned, extending the ability to apply that concept and its developmental variants to an ever widening range of alternative and varied contexts—multiple communities of practice” [24].

## Conclusions

Analysis of the data has advanced our understanding of how PAL works for health professions students in different settings (classroom and clinical) across multiple professions to enhance their **learning experience** and contribute to future career competence. The findings indicate the importance of providing appropriate training in peer teaching and feedback prior to PAL activity, which clearly demonstrated an improvement in peer tutor confidence following the PAL training. The study offers some key insights into peer tutors’ and learners perceptions’ of their PAL experiences and suggests students engagement and openness to peer learning in the classroom and clinical setting were key to the PAL activities success.

In addition, findings from this study assist in articulating how health profession educators might implement and evaluate PAL in the classroom and clinical setting. Importantly, if PAL is to be successful and sustainable as a valuable teaching and learning tool across the faculty the PAL activities must be structured, well organised and monitored to ensure students’ learning experience is optimised. Where there is a less collaborative and cooperative culture amongst students’ skill attainment may be limited.

### Limitations

It must be acknowledged that this research study was a pilot project that provided a “snapshot” of PAL activities across one faculty at one Australian university over a 14 week time period. Further research would be necessary with other health professions faculties across a larger sample of universities to determine if the findings are consistent and transferable to other health professions student populations and faculties. However, despite being relatively small, this study provides key insights into how we might begin to evaluate PAL activities in a more systematic, sustainable way and how PAL influences the students learning experience across the classroom and clinical setting.

In conclusion, the pilot PAL projects successfully addressed the project outcomes, allowing the project team to develop, implement and evaluate PAL across six courses within the FMDHS.
